# Follow-up outcome analysis of 324 cases of early-onset and late-onset mild fetal ventriculomegaly: a retrospective cohort study

**DOI:** 10.1186/s40001-024-01709-7

**Published:** 2024-02-16

**Authors:** Xuemei Wang, Shanlong Zhang, Jingjing Wang, Simin Zhang, Li Feng, Qingqing Wu

**Affiliations:** 1 Ultrasound Department, Beijing Obstetrics and Gynecology Hospital, Capital Medical University, Beijing Maternal and Child Health Care Hospital, No.251 Yaojiayuan Road, Chaoyang District, Beijing, 100026 People’s Republic of China; 2 Ultrasound Department, The Second Affiliated Hospital of Shandong First Medical University, 271000 Taian, Shandong People’s Republic of China

**Keywords:** Mild ventriculomegaly, Fetus, Ultrasound, Gestational age, Neurodevelopment

## Abstract

**Background:**

Mild fetal ventriculomegaly (VM) is a nonspecific finding common to several pathologies with varying prognosis and is, therefore, a challenge in fetal consultation. We aimed to perform a constant, detailed analysis of prenatal findings and postnatal outcomes in fetuses with early-onset and late-onset mild ventriculomegaly, and provide a new evidence basis and new perspective for prenatal counseling.

**Methods:**

This is a retrospective cohort study of women with a diagnosis of mild fetal VM between January 2018 and October 2020. The population was divided into two groups according to the gestational ages (GAs) at initial diagnosis: the early-onset group (diagnosed at/before 24^+6^ weeks) and the late-onset group (diagnosed after 24^+6^ weeks). Clinical data and pregnancy outcomes were obtained from hospital records. The children’s neurodevelopment status was assessed using the Ages and Stages Questionnaire, Third Edition (ASQ-3) and telephone interviews.

**Results:**

Our study cohort comprised 324 fetuses, out of which 94 (29%) were classified as early-onset group and 230 (71%) late-onset group. Early-onset group was more likely to have concurrent additional abnormalities, whereas in the late-onset group, isolated enlargement was more common (*P* = 0.01). Unilateral enlargement was more common in the late-onset group (*P* = 0.05), and symmetrical enlargement in the early-onset group (*P* < 0.01). In addition, early-onset mild VM cases were more likely to have intrauterine progression (*P* = 0.03), and many had a higher proportion of complex multisystem abnormalities. Compared with the late-onset group, the early-onset group was more often associated with congenital brain structure malformations. Approximately 11% of fetuses with mild VM had postnatal neurodevelopmental delay/disorders, and the risk was higher in the early-onset group (19.4% vs. 7.4%). Regression analysis showed that the GA at first diagnosis, non-isolated, and intrauterine progression significantly correlated with neurodevelopmental abnormalities.

**Conclusions:**

Early-onset and late-onset mild VM had significantly different ultrasound features and outcomes. Early-onset mild VM may have more complex potential abnormalities and are more likely to predict poor prognosis than the late-onset.

**Supplementary Information:**

The online version contains supplementary material available at 10.1186/s40001-024-01709-7.

## Background

Mild fetal ventriculomegaly (VM) refers to the abnormal fetal enlargement of the lateral ventricle (atrial width of 10–12 mm, regardless of gestational age) measured at the standard section [1]. Mild VM is a common intracranial manifestation in prenatal routine ultrasonography, with an incidence of approximately 1–22 out of 1000 live births [2–4]. Although > 90% of fetuses with mild VM have a normal postnatal evaluation, congenital structural malformations and postnatal neurodevelopmental abnormalities could still be possible [1, 5–7]. This uncertainty leads to difficulties in prenatal counseling and management and likely causes anxiety among parents. Therefore, the predictors of poor prognosis in fetuses with mild VM are highly important.

Most of the current studies have been conducted by classifying patients according to the width of the lateral ventricle or the presence/absence of concurrent additional abnormalities. Several important factors affecting the prognosis of fetuses with mild VM include the degree of VM, asymmetry, bilateral and intrauterine progression [8–11]. Considering that fetal brain structure develops throughout pregnancy, any interference from the internal or external environments of the body may cause abnormalities, which eventually affect fetal prognosis. Therefore, the gestational age (GA) at which mild VM occurs is also one of the factors that should be considered when analyzing the etiology and evaluating the prognosis. However, studies focusing on these aspects are still limited. Hence, this study aimed to analyze the prenatal ultrasound findings and postnatal outcomes of fetuses with mild VM at different GAs to provide a new evidence basis and new perspective for prenatal counseling.

## Methods

### Study population

This retrospective cohort study included pregnant women who were carrying fetuses with mild VM assessed via prenatal ultrasound at Beijing Obstetrics and Gynecology Hospital, Capital Medical University. Beijing Maternal and Child Health Care Hospital between January 2018 and October 2020.

The inclusion criteria were as follows: a fetal lateral ventricular width of 10–12 mm by prenatal ultrasound; singleton pregnancy; and GA of 15–33 weeks. Conversely, the exclusion criteria were the serious lack of imaging or clinical data and other factors affecting prognosis (preterm delivery, birth injury, etc.). GA was determined according to the start of the first day after the last menstruation and verified by ultrasound measurements in the first trimester of pregnancy.

### Fetal ultrasound

Neurosonography was performed with a multiplanar assessment according to the International Society of Ultrasound in Obstetrics and Gynecology guidelines (ISUOG 2007) [12]. The lateral ventricle was measured in the transventricular plane. The measurement was performed at the level of the glomus of the choroid plexus, perpendicular to the ventricular cavity, positioning the calipers inside the echoes generated by the lateral walls. After the first detection of mild VM by ultrasound, changes in the lateral ventricles were examined every 2–4 weeks (at least three times, except for those aborted) until birth. Senior chief physicians regularly reviewed the retained ultrasound images to determine the actual presence of abnormalities.

According to the Practice Guidelines for Performance of Prenatal Ultrasound Screening of China, each pregnant woman should undergo prenatal ultrasound screening at 20–24^+6^ weeks of pregnancy. In our hospital, women were routinely screened twice in the second trimester of pregnancy, once before 24^+6^ weeks of gestation for fetal structure screening and early growth assessment, and once after 24^+6^ weeks of gestation as a supplement to the previous screening. Through this method, we could detect more abnormalities that were blocked or not yet shown in the previous examination.

Upon reviewing the relevant literature [13, 14, 19], combining the results with our own practical experience, we classified mild VM cases into two groups: the early-onset group (diagnosed at/before 24^+6^ weeks) and the late-onset group (diagnosed after 24^+6^ weeks). First, this is the cutoff GA for two prenatal ultrasound screenings of our hospital. Second, most pregnant women included in this study used transabdominal ultrasound examination. In early pregnancy examinations, subtle structural abnormalities of the fetus were detected difficultly due to the low GAs, which affected the description and grouping of prenatal ultrasound features in cases. Third, the latest GA allowed for miscarriage in China is 28 weeks. We chose the cutoff 24^+6^ weeks, so that if abnormalities were found in the fetus, parents still had enough time to consult or consider giving up.

We recorded the detailed ultrasound characteristics and development of each case. Asymmetry was defined as a width difference of ≥ 2 mm between bilateral lateral ventricles [15, 16, 28]; otherwise, it was symmetry. Unilateral ventricular width ≥ 10 mm indicated unilateral enlargement, whereas bilateral ventricular width ≥ 10 mm indicated bilateral enlargement. In addition to the first diagnosis, VM was defined as transient when the lateral ventricle width remained < 10 mm during re-examinations, and non-transient when it persisted at ≥ 10 mm several times during the ultrasound follow-ups. Compared with the initial measurement, a ventricular enlargement range within 2 mm indicated stability, but > 2 mm indicated progression; additionally, a lateral ventricular diameter returning to < 10 mm during ultrasound follow-ups was defined as regression [17, 18]. Isolated VM (IVM) was an exclusive diagnosis. When no other abnormalities, such as structural or chromosomal abnormality or infection, were found during pregnancy, the condition was considered to be IVM; otherwise, it was a non-isolated VM.

### Prognostic analysis

On the 42nd postnatal day, all live born infants underwent physical examinations, including general physical examinations (e.g., body length and weight) and neurological examinations (e.g., reflex and muscle strength). Thereafter, an experienced ultrasound physician and a pediatrician conducted regular telephone follow-ups. The pediatrician made a structured telephone interview with the parents of each child and recorded detailed results, including their diagnosis and treatment histories, medical records, and current status. A further examination was scheduled when necessary.

During the follow-up period, all participants were asked to complete the Ages and Stages Questionnaire, Third Edition (ASQ-3). It has adequate psychological measurement characteristics and obtained high reliability and validity in China. This questionnaire, which is generally filled out by parents, can be used to screen for potential developmental problems in children aged 1–59 months. Parents need to answer 30 questions covering five areas of development, namely, communication, gross motor, fine motor, problem solving and personal social skills. Parents were asked to try various activities with their children to make an accurate assessment. Subsequently, the total score in each field was compared with the cutoff point of China’s national standards.

In this study, cases with the following conditions are referred to neurodevelopmental abnormalities: the ASQ-3 score was below the cutoff value in two or more areas; or the score was below the cutoff value in one area, and near the cutoff level in one or more areas; or identified neurodevelopmental disorders, such as intellectual disabilities, cerebral palsy, epilepsy, and so on.

### Statistical analysis

We used *t* test and Chi-square or Fisher’s exact test for analyzing continuous and categorical variables, respectively. A *P* value < 0.05 indicated a statistically significant difference. Multivariate correlation between ultrasound parameters and neurodevelopmental abnormalities was analyzed using binary logistic regression. The prenatal variables included early-onset or late-onset, isolated or non-isolated, unilateral or bilateral, symmetric or asymmetric, transient or non-transient, and intrauterine development. All statistical data were analyzed using SPSS 26.0 software.

## Results

### Patient characteristics

We included 324 (0.8% of 40,192 singleton pregnancies) cases of mild VM. Of these, 94 (29%) were in the early-onset group and 230 (71%) were in the late-onset group, with a mean GA at diagnosis of 22.96 and 29.59 weeks, respectively. Table 1 lists the participants’ baseline characteristics.

**Table 1:** Baseline characteristics of the study population

Baseline parameters	Early-onset group (*n* = 94)	Late-onset group (*n* = 230)	*P* value
Mother’s age (years)			
< 35	74 (78.7)	170 (73.9)	0.36
≥ 35	20 (21.3)	60 (26.1)	
BMI (kg/m^2^)	21.63 ± 3.72	21.69 ± 3.18	0.88
Parity			
Primipara	69 (73.4)	143 (62.2)	0.05
Multipara	25 (26.6)	87 (37.7)	
Mode of conception			
Natural	88 (93.6)	219 (95.2)	0.76
Artificial	6 (6.4)	11 (4.8)	
TORCH			
Negative	39 (92.9)	87 (87.0)	0.47
Positive	3 (7.1)	13 (13.0)	
Additional abnormalities on MRI	11 (18.3)	3 (3.3)	0.01
GA at initial diagnosis(weeks)	22.96 ± 1.18	29.59 ± 2.21	< 0.01

*BMI* body mass index, *TORCH* toxoplasmosis, rubella, cytomegalovirus, herpes simplex, and parvovirus test, *MRI* magnetic resonance imaging, *GA* gestational age

TORCH test, which assesses for the presence of toxoplasmosis, rubella, cytomegalovirus, herpes simplex, and parvovirus, was performed during pregnancy in 42 (44.7%) and 100 (43.5%) pregnant women of the early-onset and late-onset groups, respectively. Two had positive herpes simplex virus IgM, and 1 had positive *Toxoplasma* IgM in the early-onset group. In the late-onset group, 9 had positive herpes simplex virus IgM, 2 had positive rubella virus IgM, 1 had positive *Toxoplasma* IgM, and 1 had positive herpes simplex virus IgM complicated with positive parvovirus B19 IgM.

Moreover, 151 cases underwent fetal magnetic resonance imaging (MRI) examination, with 60 in the early-onset group and 91 in the late-onset group. Important information not found during the ultrasound was detected in 14 cases. Among them, early-onset (*n* = 11), non-isolated (*n* = 11), bilateral (*n* = 8), symmetric (*n* = 10), nontransient (*n* = 10), and intrauterine stable or progressive (*n* = 7) enlargement were the common ultrasound characteristics. The most common finding was corpus callosum anomalies (*n* = 7), followed by cortical dysplasia (*n* = 5), gray matter heterotopia (*n* = 3) and intracranial hemorrhage (*n* = 3). Seven cases of corpus callosum anomalies included 2 cases of dysplasia, 3 cases of hypoplasia, 2 cases of agenesis of which 1 case of partial agenesis was confirmed by autopsy after induced labor.

### Ultrasound results

Table 2 describes the prenatal ultrasound characteristics of the two groups. All the ultrasound characteristic parameters showed statistical significance among these groups. Among them, isolated and unilateral enlargement was more common in the late-onset group and symmetrical enlargement in the early-onset group. Most cases of mild VM showed non-transient enlargement, but more than half of them had regressed during pregnancy. In addition, early-onset mild VM cases were more likely to have intrauterine progression. Some cases chose to induce labor or did not undergo standardized ultrasound follow-up; thus, it was difficult to accurately judge their transient or intrauterine development, so they were eliminated during statistics.

**Table 2:** Prenatal ultrasound characteristics of mild VM in the early-onset and late-onset groups

	Early-onset group (*n* = 94)	Late-onset group (*n* = 230)	*P* value
Isolated or not			
Isolated	51 (54.3)	158(68.7)	0.01
Non-isolated	43 (45.7)	72(31.3)	
Laterality			
Unilateral	62 (66.0)	176 (76.5)	0.05
Bilateral	32 (34.0)	54 (23.5)	
Symmetry			
Symmetric	62 (66.0)	109 (47.4)	< 0.01
Asymmetric	32 (34.0)	121 (52.6)	
Transience			
Transient	26 (29.5)	97 (42.5)	0.03
Non-transient	62 (70.5)	131 (57.5)	
Intrauterine changes			
Intrauterine regression	46 (56.8)	130 (58.0)	0.03
Intrauterine progression	12 (14.8)	13 (5.8)	
Intrauterine stabilization	23 (28.4)	81 (36.2)	

In the early-onset group, approximately half of the fetuses had other structural abnormalities, whereas in the late-onset group, isolated enlargement was more common (approx. 70%). The most common additional abnormalities in both groups were abnormalities in the central nervous system (CNS), but the types varied. Several abnormalities, such as holoprosencephaly, cortical abnormalities, corpus callosum anomalies, porencephaly, cerebellar hypoplasia, Dandy–Walker malformation, and spina bifida, were noted in the early-onset group, and aqueductal stenosis and vein-of-Galen malformation in the late-onset group. Compared with the late-onset group, the early-onset group had a higher proportion of complex multisystem abnormalities (Additional file 1: Table S1).

All cases underwent the maternal serological test. We found 47 and 132 cases who underwent Down’s syndrome screening in the early-onset and late-onset groups, and 40 and 106 cases for the noninvasive DNA test, respectively. Considering the high cost of examination and the risk of the procedure, 83 cases underwent invasive examination, with 46 in the early-onset group and 37 in the late-onset group. Subsequently, 20 cases obtained abnormal results, with 9 and 11 in the early-onset and late-onset groups, respectively. In the early-onset group, 1 had trisomy 21, 1 had trisomy 18, and 7 had copy number variations (CNVs), of which 1 case had a microdeletion [seq [hg19]del (5) (p15.33p15.11)] suggesting cry du chat syndrome and another microdeletion [seq [hg19]dup (6) (p25.3p23)] in the same case, with the clinical phenotypes including microcephaly and mild developmental delay. In the late-onset group, 1 had trisomy 21, 1 had partial trisomy 22, 1 had Klinefelter syndrome, and 8 had CNVs, of which 1 case had a microdeletion [seq [hg19]del (17) (p11.2)] suggesting Smith–Magenis syndrome, which may lead to severe neurological symptoms.

### Prognostic outcomes

All fetuses were delivered in the aforementioned hospital. The pregnancy outcomes are shown in Table 3. Mild VM was more common in males, with a male-to-female ratio of 1.3 (184/140). Live birth was observed in 78 cases (83%) in the early-onset group and 225 (97.8%) in the late-onset group. The GA at delivery between the two groups were compared (*P* = 0.04). As decided by the parents, 19 cases underwent pregnancy termination, with 15 in the early-onset group and 4 in the late-onset group. In each group, one suffered from intrauterine fetal death. The autopsy results showed that the case in the early-onset group had cleft lip and palate, syndactyly involving both hands, and ectrodactyly involving both feet; in the late-onset group, the intrauterine fetal death occurred at 38 weeks of gestation, with a large number of cocci and fungal infection in the fetal blood culture, suggesting retrograde infection from the cervix.

**Table 3:** Pregnancy outcomes

	Early-onset group (*n* = 94)	Late-onset group (*n* = 230)	*P* value
Pregnancy outcomes			
Live birth	78 (83.0)	225 (97.8)	< 0.01
Intrauterine fetal death	1 (1.1)	1 (0.4)	
Termination of pregnancy	15 (15.9)	4 (1.7)	
Mode of delivery			
Vaginal delivery	50 (64.1)	130 (57.8)	0.33
Cesarean section	28 (35.9)	95 (42.2)	
GA at delivery(weeks)	38.38 ± 2.12	38.92 ± 1.30	0.04
Sex			
Male	49 (52.1)	135 (58.7)	0.28
Female	45 (47.9)	95 (41.3)	
Birth weight(g)	3386.79 ± 680.99	3449.36 ± 453.44	0.45

*GA* gestational age

All the live born infants returned to the hospital for physical examination 42 days after birth, and none of them was diagnosed with neurodevelopmental abnormalities. They were followed up for 1–4 years after birth. A total of 61 cases could not be followed up, of which 25 had a serious lack of follow-up data, thereby making it difficult to evaluate the children’s development status; for 27 cases, their parents asked to withdraw from the study because they thought that their children were in good health, and for 9 cases, their parents changed their contact information without prior notice. Finally, 242 remaining cases were followed up successfully. Although most of the 242 infants had favorable results, 19.4% and 7.4% from the early-onset and late-onset groups showed neurodevelopmental abnormalities, respectively (Table 4).

**Table 4:** Outcomes of postnatal follow-up

	Early-onset group (*n* = 78)	Late-onset group (*n* = 225)	*P* value
Normal neurodevelopment	54 (80.6)	162 (92.6)	0.007
Neurodevelopmental delay/disorder	13 (19.4)	13 (7.4)	
	Speech/language delay	Speech/language delay	
	Gross motor delay	Gross motor delay	
	Mild global development delay	Epilepsy	
	Hydrocephalus, received shunt surgery	Intellectual disability	
Lost to follow-up	11 (14.1)	50 (22.2)	

The variables with statistical variances were analyzed using binary logistic regression, and the results showed that the GA at first diagnosis, non-isolated, and intrauterine progression significantly correlated with neurodevelopmental abnormalities (Table 5).

**Table 5:** Correlation between neurodevelopmental delays/disorders and prenatal ultrasound characteristics in fetuses with mild VM

	Grouping	OR value	95% CI	*P* value
GA at the first diagnosis	Early-onset	2.864	1.141–7.101	0.025
	Late-onset^†^			
Isolated or not	Non-isolated	2.621	1.079–6.367	0.033
	Isolated^†^			
Laterality	Bilateral	2.054	0.581–7.266	0.264
	Unilateral^†^			
Symmetry	Symmetric	0.665	0.203–2.178	0.500
	Asymmetric^†^			
Transience	Nontransient	0.323	0.066–1.567	0.161
	Transient^†^			
Intrauterine changes	Progression	11.153	2.063–60.298	0.005
	Stable	2.179	0.439–10.828	0.341
	Regression^†^			

Items with ^†^ refer to the control group. *CI* confidence interval, *OR* odds ratio, *GA* gestational age

## Discussion

This study analyzed the prenatal ultrasound characteristics and prognosis of fetuses with mild VM occurring at different GAs. Prenatal ultrasound characteristics, pregnancy outcomes, and neurodevelopmental follow-up results of mild VM differed between the early-onset and late-onset groups. The outcome of the early-onset group was worse and more likely to indicate a poor prognosis than that of the late-onset group. Binary logistic regression analysis showed that early-onset mild VM, non-isolated, and intrauterine progression significantly correlated with postnatal neurodevelopmental abnormalities.

Although not usually regarded as a conclusive diagnosis, fetal mild VM may be a signal indicating potential abnormalities, such as CNS abnormalities, hypoxia, infection, and heredity. To evaluate the prognosis, we need to identify the causes of VM. In this study, 35% (115/324) of the cases had concurrent additional abnormalities, with a higher proportion in the early-onset group than in the late-onset group (46% vs. 31%). Consistent with the results of previous studies [10, 16], CNS abnormalities were the most common additional abnormalities associated with mild VM. However, the abnormal characteristics of CNS differed between the early-onset and late-onset groups, suggesting a difference in the etiology of mild VM occurring at different GAs. A recent study by Bhatia A et al. [19] also supports this perspective. Holoprosencephaly, spina bifida, Dandy–Walker malformation, and corpus callosum anomalies were common in the early-onset group, mainly associated with congenital CNS malformations. In contrast, ventricular hemorrhage, aqueductal stenosis, and vein-of-Galen malformation were more common in the late-onset group, usually considered as the secondary changes of acquired hypoxia, infection, or tumor. In addition, the incidence of complex multisystem abnormalities was significantly higher in the early-onset group than in the late-onset group.

MRI can detect 1%–17% of important additional abnormalities [20–23], but its application in fetuses with mild VM remains controversial. Some studies [24, 25] reported that for isolated VM cases, ultrasound performed by an experienced team was sufficient, and the additional contribution of MRI in such cases is limited, especially in early gestational weeks. In the present study, important information that could not be detected by ultrasound was found in 9.3% (14/151) of our cases; such information included corpus callosum anomalies, malformations of cortical development, neuron migration disorder, intracranial hemorrhage, and so on. Two cases of corpus callosum agenesis were not detected on ultrasound but found on MRI, and one of them was confirmed to be partial agenesis of corpus callosum by autopsy. We think that the reasons for missed diagnosis maybe the early gestational age (23^+4^ weeks and 24^+0^ weeks) and transabdominal examination, which affects the operator’s observation quality of fine structures. A recent international multicenter study [23] has shown that the diagnostic value of MRI decreased when a multiplanar ultrasound examination of the fetal brain was performed. However, based on the special value of MRI in detecting migration disorders and hemorrhage, MRI assessment is still supported in every fetus with a prenatal diagnosis of VM. Another multicenter study [26] suggested that, even in cases of isolated mild VM, there was a 1:20 chance that additional brain abnormalities had been overlooked on ultrasound; thus, they believed that MRI should be offered to all VM cases for further evaluation. Based on our data, we support the above viewpoints. Considering the limitation in the penetration of the fetal skull and operation techniques, these fetal CNS abnormalities, such as cortical malformations, migration disorders and hypoplasia or partial agenesis of the corpus callosum, often cannot be detected by ultrasound, but can be displayed on MRI. We opine that MRI should be used as a supplementary examination tool for fetuses with mild VM to minimize missed and delayed diagnosis.

VM, as a soft ultrasound remark, can increase the risk of fetal chromosome abnormalities; thus, the karyotype should be analyzed regardless of the degree of dilation or concurrent additional abnormalities [27, 28]. Our study supports the above view. In this study, 20 cases were found to have abnormalities, including trisomy 21, trisomy 18, partial trisomy 22, Klinefelter syndrome, and CNVs in 2, 1, 1, 1, and 15 cases, respectively, and 2 CNVs were reportedly associated with intellectual disability or developmental delay. Compared with conventional karyotyping, chromosomal microarray analysis (CMA) improves the prenatal detection rates and can identify additional, clinically significant cytogenetic information in fetuses with CNS anomalies and a normal karyotype [29]. Therefore, it is recommended that CMA be used for prenatal diagnosis and karyotype analysis of mild VM fetuses. Invasive examination significantly increased the detection rate of fetal chromosome abnormalities in mild VM. However, we found that when VM was considered isolated or not severe, the surgical risk and high price of invasive examination affected parents’ choice.

Further, fetal VM may be associated with infection during pregnancy, particularly cytomegalovirus and *Toxoplasma gondii* [1, 30, 31]. Considering that the infection rate in fetuses with VM has been reported to be very low (1.5–4.5%), infection screening is not routinely performed. Thus, data supporting the aforementioned view remain insufficient. In our study, TORCH screening for pregnant women was conducted in 142 cases in early pregnancy. Positive IgM was found in 16 cases, with a higher rate in the late-onset group than in the early-onset group (13/100 vs. 3/42). Different from the results of previous studies, the positive rate of herpes simplex virus IgM was the highest (12/16) in all of the IgM-positive cases. However, we cannot draw any conclusions because the fetal infection was not further confirmed for these 12 pregnant women. Fetal VM is reportedly a manifestation of intrauterine transmission of the herpes simplex virus [32]. This view needs to be further studied and verified in the future.

VM in early pregnancy has been recently reported to be a significant factor in poor prognosis for fetal survival after birth [33]. In this study, the live birth rate of fetuses with VM was significantly higher in the late-onset group, and more parents in the early-onset group chose to terminate the pregnancy. All the fetuses aborted in this study had other serious structural malformations or chromosome abnormalities. Therefore, the incidence of concurrent additional abnormalities in the two groups is the primary reason for the above mentioned differences. Notably, the late-onset group still has risks of concurrent additional abnormalities and postnatal neurodevelopmental delays; hence, the parents should be informed of these risks during prenatal counseling and followed up after birth.

Many studies have evaluated the neurodevelopment of children experiencing prenatal mild VM, but the reported results vary highly because of differences in the study population, grouping criteria, postnatal development assessment methods, and follow-up period. We have studied the prevalence of neurodevelopmental delay in children born with mild VM in the literature and discovered that a normal developmental outcome was found in more than 90% of cases [1, 6, 34]. The ASQ-3 has long been used in the field of neurodevelopment, and its reliability and validity has been validated in many different populations and languages [35–37]. According to a systematic review [37], the sensitivity and specificity of the ASQ-3 are reliable compared with the Bayley scales, which is considered the gold standard for assessing infant development. However, the ASQ-3 is a developmental screening tool, rather than a diagnostic tool, which has a relatively high false positives rate. Thus, we used a strict definition of abnormality to minimize this potential bias. In this study, approximately 11% (26/242) of fetuses with mild VM had postnatal neurodevelopmental disorders, consistent with the previous studies [31, 38, 39]. Upon comparison between the two groups, the risk of postnatal neurodevelopmental disorders was higher in the early-onset group (19.4% vs. 7.4%).

In our multivariate regression analysis, early mild VM, non-isolated, and intrauterine progression were risk factors for postnatal nervous system dysplasia. Although GA at the onset of mild VM cannot be informative by itself to predict neurodevelopmental disorders, it can indicate possible causes that maybe an important determinant of postnatal outcomes.

One of the strengths of this study is that it is one of the few studies specifically comparing the correlation between fetal neurodevelopmental outcomes and prenatal ultrasound features particularly for cases with mild VM. Another strength is that it is one of the few that includes GA at initial diagnosis as an important factor in the etiological analysis and prognosis evaluation of fetal mild VM. Moreover, we collected detailed prenatal data of all cases and conducted a detailed comparison. Further, we collected short-term birth results as well as long-term neurodevelopmental outcomes.

This study has some limitations. First, a significant limitation of this study is that follow-ups were conducted through telephone interviews and screening questionnaires. The follow-up data should be viewed with caution because the parents could not be objective. Second, it was a study reporting observational results from a single institution, but we are a national prenatal consultation center and included a large sample size. Third, the study is retrospective in nature, and not all cases underwent tests for MRI, cytogenetics, and serology, which may result in selection bias. It also lacks postnatal imaging evaluation data.

## Conclusions

Early-onset, non-isolated, and intrauterine VM progression are risk factors for postnatal nervous system dysplasia. Early-onset mild VM may have more complex potential abnormalities and are more likely to predict poor prognosis than the late-onset ones. The GA at the initial diagnosis of mild VM could be related to its etiology; thus, it should be considered in prenatal counseling and fetal management.

### Supplementary Information

**Additional file 1: Table S1.** Relevant abnormalities in prenatal findings.

## Data Availability

The data sets used and/or analyzed during the current study are available from the corresponding author on reasonable request.

## Abbreviations

ASQ-3: Age and Stages Questionnaire, Third Edition
BMI: Body mass index
CNS: Central nervous system
CNV: Copy number variations
CMA: Chromosomal microarray analysis
GA: Gestational age
MRI: Magnetic resonance imaging
OR: Odds ratio
TORCH: Toxoplasmosis, rubella, cytomegalovirus, herpes simplex, and parvovirus test
VM: Ventriculomegaly
