# Supplementary material for: Follow-up outcome analysis of 324 cases of early-onset and late-onset mild fetal ventriculomegaly: a retrospective cohort study
Source: Eur J Med Res. 2024 Feb 16;29:128. doi: 10.1186/s40001-024-01709-7 (PMC10870476; doi:10.1186/s40001-024-01709-7)
Supplement: Supplementary file 1 — Additional file 1: Table S1. Relevant abnormalities in prenatal findings. [file 40001_2024_1709_MOESM1_ESM.docx]

**Additional file 1: Table S1** Relevant abnormalities in prenatal findings

| Relevant abnormalities | Early-onset group (n = 43/94; 45.7) | Late-onset group (n = 72/230; 31.3) |
| --- | --- | --- |
| CNS | (n = 18; 41.9) | (n = 32; 44.4) |
|  | Holoprosencephaly | Cortical abnormalities |
|  | Cortical abnormalities | Corpus callosum anomalies |
|  | Corpus callosum anomalies | Ventricular hemorrhage |
|  | Porencephaly | Abnormal echoes in choroid plexus |
|  | Cerebellar hypoplasia | Periventricular leukomalacia |
|  | Dandy–Walker malformation | Aqueductal stenosis |
|  | Spina bifida | Vein-of-Galen malformation |
|  | Intraparenchymal hemorrhage | Septo-optic dysplasia |
|  | Ventricular hemorrhage | Arachnoid cyst |
|  | Abnormal echoes in choroid plexus | Dilated cisterna magna |
|  | Periventricular leukomalacia |  |
|  | Agenesis/ hypoplasia of septum pellucidum |  |
|  | Arachnoid cyst |  |
|  | Dilated cisterna magna |  |
| Non-CNS | (n = 8; 18.6) | (n = 18; 25.0) |
|  | Tetralogy of Fallot | Tetralogy of Fallot |
|  | Ventricle septal defect | Ventricular septal defect |
|  | Renal pelvis dilatation | Vascular ring in the heart (RAA + LDA + ALSA) |
|  | Cystic hygroma in the neck | Multicystic dysplastic kidney |
|  |  | Renal pelvis dilatation |
|  |  | Increased liver echogenicity |
|  |  | Dilated/echogenic bowel |
|  |  | Increased NF thickness |
| Multisystem abnormalities | (n = 5; 11.6) | (n = 0; 0.0) |
|  | Abnormal limbs, megacystis, and arrhythmia |  |
|  | Gray matter heterotopia, dilated cisterna magna, ocular hypotelorism, and FGR |  |
|  | Corpus callosum hypoplasia, cortical abnormalities, Dandy–Walker malformation, polydactyly involving both hands, and polydactyly involving both feet |  |
|  | Intraventricular hemorrhage, cerebral edema, Tetralogy of Fallot, and FGR |  |
|  | Polydactyly involving both hands, polydactyly and syndactyly involving right foot, and polyhydramnios |  |
| Polyhydramnios/oligohydramnios  Chromosome abnormalities | (n = 3; 7.0)  (n = 9; 20.9)  Trisomy 21  Trisomy 18  Copy number variations | (n = 11; 15.3)  (n = 11; 15.3)  Trisomy 21  partial Trisomy 22  Klinefelter syndrome  Copy number variations |

CNS, central nervous system; NF, nuchal fold; RAA + LDA + ALSA, right aortic arch with left-sided ductus arteriosus and aberrant left subclavian artery; FGR, fetal growth restriction.
